# Drug Delivery in Photodynamic Therapy

**DOI:** 10.3390/pharmaceutics15071784

**Published:** 2023-06-21

**Authors:** Francesca Moret, Greta Varchi

**Affiliations:** 1 Department of Biology, University of Padova, 35100 Padova, Italy; 2 Institute for the Organic Synthesis and Photoreactivity, Italian National Research Council, 40121 Bologna, Italy

Photodynamic therapy (PDT) has gained prominence as a non-invasive and selective treatment option for solid tumors and non-oncological diseases. However, limitations such as the shallow penetration of light into tissues and poor bioavailability of photosensitizers (PS) hinder its efficacy. To address these challenges, researchers are exploring nanotechnology-based delivery vehicles and cell-based approaches to improve PS distribution, targeted accumulation, and controlled drug release. This Special Issue showcases the advancements in drug delivery systems for oncological and non-oncological PDT.

This editorial aims at giving an overview of the eight research articles and the seven review papers that were published in the Special Issue.

Obaid and colleagues focused their study on improving the performance of an Osmium (II)-based photosensitizer (ML18J03) that was formulated into DSPE-mPEG2000 micelles. This formulation not only improved the luminescence of the photosensitizer but also increased its tumor selectivity. By encapsulating the photosensitizer within micelles, researchers were able to enhance its accumulation in tumor tissues and achieve a higher level of selectivity, addressing the challenge posed by the photosensitizer’s low-luminescence quantum yield [1].

Combination therapies have been gaining attention as a means to augment the therapeutic outcomes of cancer treatments. In this context, Duchi and collaborators explored the co-encapsulation of Chlorin-e6 (Ce6) and paclitaxel (PTX) within keratin nanoparticles for the treatment of osteosarcoma (OS). This combination showed promising results in inhibiting tumor cell growth. By delivering both Ce6 and PTX together, the researchers observed a synergistic effect in an orthotopic model of OS that led to a significant reduction in tumor size compared to using either therapy alone [2].

Muragaki and coworkers analyzed the efficacy of talaporfin sodium-mediated PDT as a treatment for recurrent glioblastoma (GMB). A retrospective analysis was conducted on 70 patients who underwent surgery with PDT and 38 patients who had surgery alone. The results showed that the PDT group had a longer median progression-free survival compared to the control group. The median overall survival after the second surgery was also longer in the PDT group. The analysis further revealed that the efficacy of PDT was consistent regardless of the pre-recurrence pathology, indicating potential survival benefits for recurrent GBM patients [3].

On the same topic, Tsung Yang and colleagues focused on the development of new therapeutic options for treating GMB. The authors studied the use of photochemical internalization to release therapeutic drugs into GBM cells using light-activated photosensitizers. The study employed etoposide (Etop) and protoporphyrin IX (PpIX) loaded into polyamidoamine dendrimers nanospheres. This formulation showed enhanced cellular uptake compared to free PpIX, and light irradiation resulted in increased synergistic effects, oxidative stress, and apoptosis compared to treatment with Etop and PpIX alone [4].

In an effort to address the challenge of precise drug localization in cancer therapy, the study by Nonell and coworkers focused on developing targeted chemo-photo-nanocarriers. These nanocarriers were designed to target cells overexpressing the epidermal growth factor receptor (EGFR) and release the chemotherapeutic agent doxorubicin upon exposure to light. The study found that cetuximab-IRDye700DX-mesoporous silica nanoparticles loaded with doxorubicin resulted in efficient and selective killing of EGFR-expressing cells. The doxorubicin was released from the nanocarrier mainly through singlet oxygen-induced mechanisms, without any toxicity in the absence of light, proving the effectiveness of this novel nanosystem for the controlled release of doxorubicin [5].

A recent study by Miolo and coworkers investigated the development of PDT-enabled delivery systems for bladder cancer treatment. Three different porphyrinic photosensitizers (TMPyP, Zn-TMPyP, and P1-C5) were loaded onto graphene oxide (GO) or graphene quantum dots (GQDs) in a one-step process. The cytotoxic effects of the free photosensitizers and their hybrids were compared under light exposure on T24 human bladder cancer cells. Results showed that the photosensitizers induced apoptotic cell death through lysosomes damage, and confirmed that immobilizing the photosensitizers onto graphene-based nanomaterials did not compromise the in vitro activity, with Zn-TMPyP@GQDs showing the most promising results upon red light irradiation [6].

PDT shows limited efficacy as a standalone treatment for solid tumors, while immunotherapy faces challenges due to the immunosuppressive state of advanced tumors. To address this issue, the Ossendorp group employed a combination of PDT and immunostimulatory nanoparticles (NPs) loaded with Toll-like receptor agonists and a lymphocyte-attracting chemokine. This combination demonstrated strong anti-tumor responses, including an abscopal effect, in three relevant mouse models of cancer. The combination treatment induced tumor-specific CD8+ T cell immune responses against tumor-specific epitopes, suggesting PDT as an in situ vaccination strategy. The treatment also transformed the tumor microenvironment from immunosuppressed to pro-inflammatory, supporting the combination of PDT with immunostimulatory NPs as a potential strategy for treating solid tumors [7].

In view of combining different therapeutic approaches in the fight against cancer, the study by Varchi and colleagues focused on developing tumor microenvironment (TME)-responsive nanoparticles exclusively composed of a paclitaxel (PTX) prodrug and the photosensitizer pheophorbide A (PheoA). When exposed to TME-mimicking conditions, the nanoparticles rapidly disassembled, leading to the release of PTX and PheoA, resulting in increased cytotoxicity. Notably, in tests with SK-OV-3 cells, the nanoformulation enabled a significant reduction in PTX and PheoA doses while maintaining efficacy. The study demonstrated that these prodrug-based nanocarriers have potential as effective and safe drug delivery systems, with the ability to reduce toxicity and facilitate translation to preclinical applications [8].

Among the review papers submitted to this Special Issue, the work by Calvaresi and colleagues presents an insightful picture of EGFR-targeted PDT, highlighting the advantages and pitfalls of this approach. The authors conclude that PDT holds promise in treating EGFR-positive cancers by utilizing EGFR as a docking mechanism to deliver photosensitizers to cancer cells. To face the issue of limited light penetration in the body, the authors also describe the use of near-infrared light-excited PSs and sonodynamic therapy (SDT) to improve tumor ablation [9].

Four reviews focus on the use of nanotechnology-enabled combination therapy for cancer treatment. Combining PDT with other treatments, utilizing nanoparticles for drug delivery, and leveraging biomimetic nanotechnology are all promising strategies for advancing cancer therapies. The specific focus on esophageal cancer highlights the need for early diagnosis and effective treatment options [10]. The reviews published on this topic highlight that nanotechnology-driven advancements hold great potential for personalized and targeted cancer treatment [11,12,13].

PDT has been discussed as an effective anti-tumor therapy using oxidative stress a as tumor cell-killing effector. However, PDT can face resistance from pre-existing or stress-induced factors, which reduces the treatment efficacy. The review by Korbelik and coworkers points to the role of low-flux NO generated by tumor cell iNOS in PDT’s acquired resistance and in the enhanced proliferative and migratory aggressiveness of cells that can withstand the photooxidative challenge. This literature survey highlights that NO from PDT-targeted cells can induce iNOS/NO in non-targeted bystander cells, making them more aggressive through a NO “feed-forward” process. The authors finally suggest that concerns about more aggressive and possibly more metastatic phenotypes of PDT-surviving cells could be mitigated by using inhibitors of iNOS enzymatic activity or iNOS transcription [14].

Only one review paper dealing with antimicrobial PDT (aPTD) was reported by Wah Wong and collaborators [15]. The work focuses on fungal keratitis, a dangerous and hard-to-treat eye infection that can cause blindness. One promising treatment option is represented by aPDT using riboflavin and Rose Bengal. However, resistance to drugs is an issue, and better drug delivery is necessary to achieve successful treatment. Recent studies have demonstrated that nanoparticles can efficiently treat melanoma cells and offer simultaneous imaging and therapy; this technology could be utilized to address the problem of inadequate drug penetration in the treatment of fungal stromal keratitis.

These studies highlight significant advancements in photodynamic therapy and combination therapies for cancerous and non-cancerous treatment. The findings provide valuable insights into improving treatment outcomes, enhancing selectivity, reducing toxicity, and addressing challenges in precise drug localization and the immunosuppressive state of advanced tumors.
